# Mapping the Incidence of Dengue Fever in the State of Pará, Eastern Amazon: Epidemiology and Relationships with Climate

**DOI:** 10.3390/reports8020061

**Published:** 2025-05-03

**Authors:** Emilene Monteiro Furtado Serra, Douglas Batista da Silva Ferreira, João de Athaydes Silva Jr, Bergson Cavalcanti de Moraes, Aline Maria Meiguins de Lima, Brenda Caroline Sampaio da Silva, Bruno Spacek Godoy, Eliane de Castro Coutinho, Andressa Tavares Parente, Julia Clarinda Paiva Cohen, Alan Cavalcanti da Cunha, Everaldo Barreiros de Souza

**Affiliations:** 1 Programa de Pós-Graduação em Ciências Ambientais (PPGCA) Programa de Pós-Graduação em Gestão de Risco e Desastre na Amazônia (PPGGRD), Instituto de Geociências, Universidade Federal do Pará, Belém 66075-110, PA, Brazil; emilene.serra@ig.ufpa.br (E.M.F.S.); athaydes@ufpa.br (J.d.A.S.J.); bergson@ufpa.br (B.C.d.M.); ameiguins@ufpa.br (A.M.M.d.L.); brendacaroline444@gmail.com (B.C.S.d.S.); jcpcohen@ufpa.br (J.C.P.C.); bspacek@ufpa.br (B.S.G.); 2 Instituto Tecnológico Vale, Belém 66055-090, PA, Brazil; douglas.silva.ferreira@itv.org; 3 Instituto Amazônico de Agriculturas Familiares, Universidade Federal do Pará, Belém 66077-530, PA, Brazil; 4 Centro de Ciências Naturais e Tecnologia, Universidade do Estado do Pará, Belém 66095015, PA, Brazil; elianecoutinho@uepa.br; 5 Instituto de Ciências da Saúde, Universidade Federal do Pará, Belém 66075-110, PA, Brazil; andressatp@ufpa.br; 6 Departamento de Engenharia Civil, Universidade Federal do Amapá, Macapá 68900-070, AP, Brazil; alancunha@unifap.br

**Keywords:** dengue, Amazon, rainy season, correlation, epidemiology, cluster-based regression

## Abstract

**Background:** The Amazon region possesses vast natural and anthropogenic ecosystems within a hydroclimatic environment conducive to the proliferation of arboviruses associated with infectious diseases in the human population, notably dengue fever, which poses a recurrent and significant public health challenge. **Objective and Methods**: We wished to update the dengue mapping for the state of Pará (eastern Amazon) using municipality-level secondary data between 2010 and 2024, including epidemiological information. Furthermore, the seasonal effects of soil and atmospheric meteorological variables (ERA5 reanalysis) on the annual municipal incidence of dengue were statistically analyzed through correlation and cluster-based regression methods. **Results**: Dengue mapping identified key areas over the central, southwest, and southeast parts of Pará, with eleven municipalities exhibiting extreme dengue counts exceeding 300 cases per 100,000 inhabitants. The epidemiological profile in these cities with worsening transmission showed a higher incidence in adults aged 20–39 years old (39%) and a predominance among women (54%). The majority of dengue cases occur during the rainy season (January to May), accounting for 69% of annual cases, when the climate conditions maximize vector proliferation. The statistical analyses highlighted the significant and spatially heterogeneous influence of regional climate variables on the dengue transmission cycle. **Conclusions**: This study advances our understanding of climatic drivers of dengue in the Amazon and provides relevant evidence to support region-specific surveillance and control strategies.

## 1. Introduction

The Amazon region has vast forested areas and natural/anthropized ecosystems in a hydroclimate environment that provides favorable conditions for the existence of several arboviruses linked to infectious diseases in the human population [1]. After malaria [2,3], dengue fever is one of the most common arboviruses spread by mosquito vectors, whose yearly recurring epidemic periods have a major negative influence on public health throughout the region [4,5].

Among the multiple factors that contribute to the transmission of dengue, involving socioeconomic, ecological, and environmental aspects, the latter are strongly related to climatic conditions, interfering in the reproductive cycle of its disease vectors [6]. The local climate conditions exert direct influences on the biology of the vector mosquito *Aedes aegypti* during its aquatic phase (eggs, larvae, and pupae in water breeding sites on the surface and in containers) and terrestrial phase for adult mosquitos in the atmospheric environment [7,8]. Morin [9] described the complexity involved in the dynamic cycle of dengue transmission, involving processes of interaction between the vector (mosquito) and regional climate factors. Thermal conditions influence the development of the vector such that high temperatures interfere with its reproduction rate and survival and the extrinsic incubation period of the virus, which speeds up the virus’s life cycle and viral replication, consequently increasing the transmission potential, as also reported by Mordecai [10]. The occurrence of precipitation directly contributes to the establishment of breeding sites and habitats suitable for mosquito reproduction, such as stagnant water on surfaces and in containers. The combination of these climatic elements can have synergistically positive effects on dengue transmission. In general, periods of abundant precipitation and warmer-than-average temperatures are often associated with peaks in dengue incidence in the subtropical and tropical regions of Brazil, including the Amazon [7,11,12].

The above-mentioned studies have addressed the effects of climate in one or more Amazonian cities where there have been dengue outbreaks by using the variables of precipitation and air temperature. A knowledge gap is the absence of studies including analyses of soil temperature and surface water conditions. Here, we use the ERA5 reanalysis [13] containing data on soil water volume and temperature (in the surface layer between 0 and 7 cm) with the intention of expanding the scientific scope of the research and providing a more complete understanding of both the soil and air climate variables that interfere in the aquatic and terrestrial/atmospheric phases of the dengue-transmitting mosquito.

In the systematic review conducted by Viana and Ignoti [7] on the occurrence of dengue and meteorological variations in Brazil, a common result is worsening of the disease being associated with increased rainfall and temperature variations, especially in the first half of each year. In a heatmap of monthly cases in 2001–2016 for each state, Churakov [14] demonstrated that dengue fever peaks between January and June in most of the Brazilian territory. In the present study, our focus is on the seasonality of dengue in eastern Amazonia. Despite constant measures to control the *Aedes aegypti* vector to minimize arboviruses, the incidence of dengue has increased in all Amazonian states from 2005 onwards, with an aggravating factor being the positive trend in fatality rates [15]. Therefore, the objective of the work is to update the dengue mapping (by official confirmed cases by the Brazilian Ministry of Health) for the state of Pará (eastern Brazilian Amazon) using municipality-level secondary data between 2010 and 2024, including epidemiological information in critical areas of disease transmission. Furthermore, the seasonal effects of soil and atmospheric meteorological variables (ERA5 reanalysis) on the annual municipal incidence of dengue were statistically examined through correlation and cluster-based regression methods. Our goal is to show that in the rainy season, the dynamics of dengue transmission are maximized over a few months, which poses a major and yearly public health concern at the municipal level.

## 2. Materials and Methods

Official data on confirmed cases of people being affected by dengue were extracted from the Department of Information Technology of the Unified Health System (DATASUS: https://datasus.saude.gov.br, accessed on 20 January 2025), coordinated by the Brazilian Ministry of Health [16]. The online version of TABWIN within DATASUS provides data processed by the Notifiable Diseases Information System (SINAN), where we obtained quantitative data on notifications of dengue cases filtered by a final classification of classic dengue and complications of hemorrhagic fever, excluding ignored/blank (no information) and discarded, inconclusive cases. The annual totals of dengue case notifications were organized for all municipalities in the state of Pará, covering a 15-year period, from 2010 to 2024.

The annual data (2010 to 2024) on the population (total number of residents) in the municipalities of the state of Pará were acquired from the Brazilian Institute of Geography and Statistics [17]. The land use and landcover (LULC) map produced in 2020 by the IBGE Environmental Coordination was downloaded to characterize the study area in the state of Pará [18].

Completing the database, we used ERA5, the fifth and most current generation of the reanalysis series developed by the European Centre for Medium-Range Weather Forecasts (ECMWF), described in detail by Hersbach [13]. ERA5 was generated using state-of-the-art data assimilation by combining observations from multiple sources (from in situ measuring stations of the global monitoring network and data derived from remote sensing using environmental satellites) that are processed in the operational numerical meteorological model. Due to its consistency and global coverage of detailed information on the state of the atmosphere, land surface, and oceans at high spatial and temporal resolutions, this database is widely used in scientific research as an essential tool for investigations addressing climate dynamics and the behavior of atmospheric variables and environmental studies. For the present study, the variables precipitation (P), air temperature (Ta) 2 m near the surface, and soil temperature (Ts) and water volume (Ws) in the layer between 0 and 7 cm (level 1) were extracted for the georeferenced grid points in each municipal seat of the state of Pará. The units for P are mm; for Ta and Ts, they are °C; and for Ws, they are m^3^/m^3^. These data were obtained as monthly averages for January to December from 1991 to 2024. The period from 1991 to 2020 was used in the calculations of the climatological average (which requires a historical series of 30 years) to define the seasonal regimes in the state of Pará.

The methodological procedures adopted in this work are described below.

**Figure 1:**
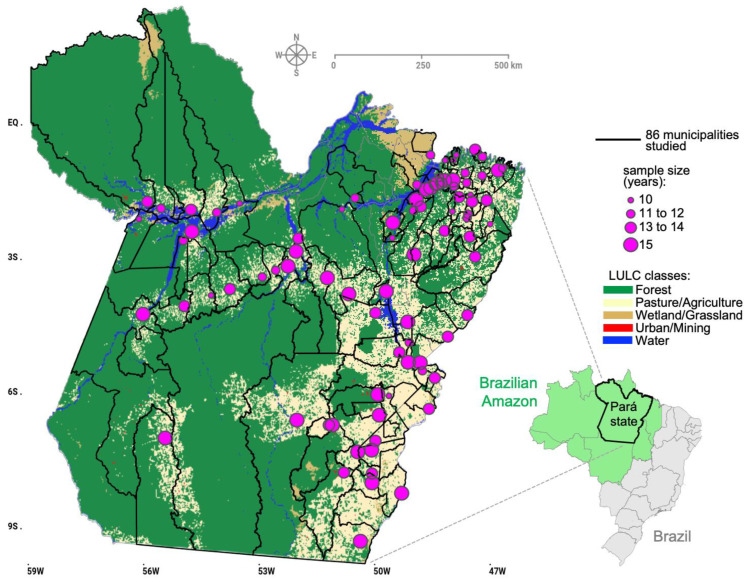
The study area, encompassing the municipalities of the state of Pará, with the LULC map in the background. Circles in each municipal seat indicate the sample size with available dengue data.

Spreadsheets with information on the annual total number of confirmed dengue cases (DATASUS) for each of the 144 municipalities in the state of Pará were created between 2010 and 2024. In this data exploration, several cities presented missing information in successive years, so only municipalities with at least 10 to 15 years of data were selected, resulting in a sample of 86 municipalities. Figure 1 illustrates the map of the study area with the geographic domain of the municipalities throughout the state and the location of the municipal seats, with which it is possible to verify the sample size (years) of the dengue data from 2010 to 2024. With an area of around 1.25 million km^2^, Pará is the largest state in the eastern Amazon, and in the LULC map, it is clear that cities have developed in anthropized areas, with the conversion of forest cover into large urbanized and agricultural areas (Figure 1). The area covered by the 86 municipalities to be analyzed corresponds to approximately 73% of the state total.

The following formula was used to determine the Annual Dengue Incidence Rate (IDengue) using the population data (IBGE) for the municipalities of Pará (IBGE):

IDengue = (annual total of new confirmed cases ÷ total population in the respective year) × 100,000(1)

The results are interpreted on spatial maps, with the scale of analysis being the municipalities of the state of Pará. The World Health Organization (WHO) recommends collecting data on confirmed cases and calculating IDengue to monitor the disease burden and guide public health policies in its epidemiological surveillance protocols for dengue [19,20].

The monthly climatological averages (for 30 years, from 1991 to 2020) for the ERA5 variables P and Ta were computed for these 86 municipalities in the state. We also obtained the monthly averages from 2010 to 2024 of the numbers of positive dengue cases for the list of these municipalities from DATASUS. Graphs of P, Ta, and dengue cases from January to December were plotted for the analysis of the annual climatological cycle. The annual average P (across all months and all municipalities) was used as a threshold to distinguish the seasonal regimes. The rainy regime (RAINY) is indicated by five consecutive months with P values above the annual average, and the dry regime (DRY) is shown by seven consecutive months with P values below the annual average. Lastly, the time series for the ERA5 variables P, Ta, Ts, and Ws for the 86 municipalities of Pará were computed in the RAINY regime from 2010 to 2024, whose data were used in the statistical analyses.

The statistical methodology used to respond to the objectives proposed in this work is described below.

The seasonal series of P, Ta, Ts, and Ws for the RAINY regime were correlated with the IDengue annual series for all municipalities from 2010 to 2024. Maps showing the spatial distribution of positive and negative correlations across the state of Pará are used to explain the results. The hypothesis that local climate variables have a significant relationship with disease incidence is adopted; that is, conditions of increased rainfall and soil water availability, along with higher air and soil temperatures, tend to favor the existence of more surface water habitats for mosquito development and an activated life cycle, which strengthens the transmission dynamics of positive dengue cases, thus increasing its incidence. These aspects were demonstrated in the scientific review conducted by [7] for Brazil and in the retrospective study by [8] evaluating the dengue cases and climate conditions in Brazilian regions. Therefore, considering these scientific aspects, positive correlations between climate and dengue are expected. Thus, the following quantitative criteria will be used to evaluate whether this hypothesis is accepted:

i. The group of municipalities with positive values (≥0.1) on the correlation maps must be significantly higher than the group with negative values (≤−0.1);
ii. To evaluate the representativeness of each group, the percentage proportions of the two groups (relative to the entire sample of municipalities = 86) are compared using the chi-square test; i.e., the difference in the proportion of the group with positive correlations must exhibit a statistically significant test value at the 5% level (*p*-value < 0.05).

The acceptance hypothesis is valid if criteria i and ii are met; otherwise, the hypothesis that there is a spatially significant relationship between climate and dengue incidence is rejected. Even though the correlation map results consider thresholds of ±0.1 (including values that are not necessarily individually significant), the combined use of the chi-square statistical test guarantees the robustness and spatial representativeness of the state’s municipal domains, thereby quantitatively validating the initial research hypothesis. In bivariate analyses, the chi-square test is frequently used to compare groups and examine proportions. Similar to our contingency analysis comparing the proportions of the correlation groups, Stevicet [21] employed this process to analyze the distributions of the responses (or proportions) between groups. We use Spearman’s correlation coefficient (ρ), which measures the monotonic relationship (increasing or decreasing trend) between the variables, as it is less sensitive to outliers and does not require the assumption of linearity. Previous studies analyzing climate and dengue occurrence data have reported close associations between climate variability and disease incidence [12,22,23].

To determine the seasonal contribution of the climate variables of the RAINY regime to the annual incidence of dengue across the state’s municipalities, we used the regression analysis approach, considering the dependent variable *y* (IDengue) and the independent or explanatory variable *x* (P, Ws, Ta, and Ts), for the period 2010 to 2024. When choosing the type of model, we performed exploratory analyses of the databases and tested linear regression and regression using mathematical transformations (e.g., logarithmic), but the results were not satisfactory. In the search for information from previous scientific studies, we found evidence of spatiotemporal patterns of clusters of dengue outbreaks occurring throughout the Brazilian territory, which were diagnosed using nonparametric estimators and bivariate spatial statistics [15,24]. Considering this context, we opted to use a cluster-based regression analysis [25] with interaction effects between *x* and the clusters in order to evaluate how the variable *x* affected *y* within each cluster and to identify whether the effects (slopes and intercepts) differed between the groups. Before, standardization (scaling) was applied to the variables to ensure comparability, and a k-means clustering algorithm with k = 3 (i.e., three clusters) was used. This assigned each observation in the dataset a cluster label that was later used as a categorical variable in the regression. After this, a multiple linear regression model was fitted with an interaction term between *x* and the clusters to allow for different intercepts and slopes for each group. The model is expressed as

*y* = β_0_ + β_1_ *x* + β_2_ C_2_ + β_3_ C_3_ + β_4_ (*x* • C_2_) + β_5_ (*x* • C_3_) + ε (2)

where C_2_ and C_3_ are indicator variables for clusters 2 and 3, respectively (with cluster 1 as the reference category). β_0_ is the intercept for cluster 1 (reference), and β_1_ represents the slope of *x* for cluster 1. β_2_ and β_3_ capture the differences in the intercepts for clusters 2 and 3, respectively, relative to cluster 1. β_4_ and β_5_ capture the differences in the slope of *x* for clusters 2 and 3, i.e., the effects of the interaction, and ε is the error or residual assumed to be normally distributed with mean zero and constant variance. In the interpretation of the coefficients for clusters 1, 2, and 3, we have the respective equations:

*y* = β_0_ + β_1_ *x* + ε (3)

*y* = (β_0_ + β_2_) + (β_1_ + β_4_) *x* + ε (4)

*y* = (β_0_ + β_2_) + (β_1_ + β_4_) *x* + ε (5)

This model allows both the intercepts and slopes to vary across clusters, thus determining the effects of *x* on *y* in each group. The model was applied separately for each climate variable P, Ws, Ta, and Ts to identify the effects of each one on the incidence of the disease. The results of the models are interpreted in terms of the scatterplots, with regression lines fitted to each cluster with intercepts and R^2^ representing how much *x* quantitatively explains the behavior of *y*, as well as analyses of the residual standard error and F-statistics considering a *p*-value < 0.05. Plotting the diagnostic graphs of histograms and Q-Q plots allowed us to check the normality of the residuals in each model.

## 3. Results

### 3.1. Mapping Dengue and the Epidemiology of Critical Areas

**Figure 2:**
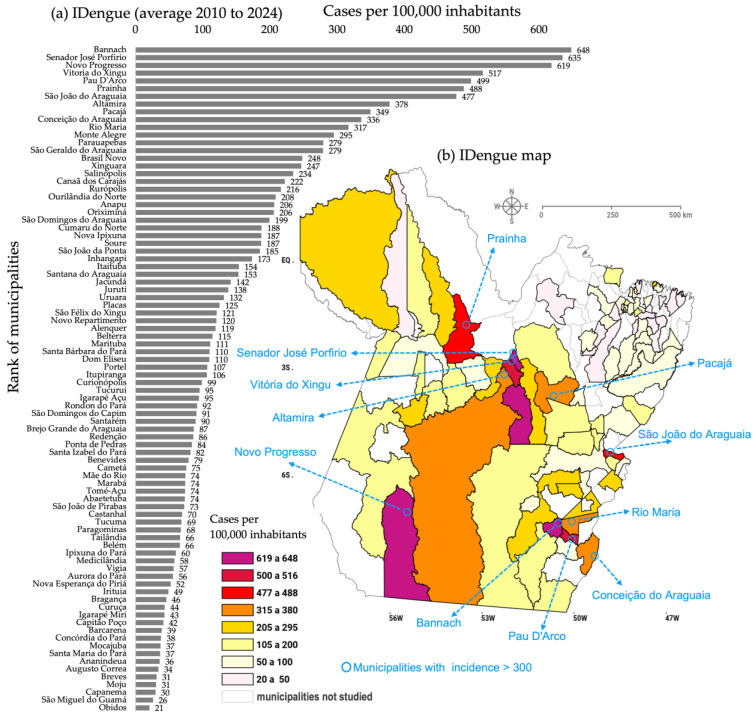
IDengue (cases/100,000 inhabitants) in the 86 municipalities of the state of Pará considering (**a**) a bar graph with the ranking and (**b**) a map with the geographic locations of the municipalities. The arrows highlight the cities with the most critical disease transmission.

The results of IDengue (average for 2010 to 2024) are shown in Figure 2 with a bar graph detailing the municipalities’ rankings, together with a map displaying the geographic locations of the municipalities throughout the state of Pará. The spatial representation corresponds to 60% (86 out of a total of 144 municipalities) of the state being affected by officially confirmed cases of dengue over the last decade and a half. There are municipalities presenting extreme values, with rates of around 600 in Bannach, Senador José Porfirio, and Novo Progresso; around 500 in Vitoria do Xingu and Pau D’Arco; approximately 480 in Prainha and São João do Araguaia; and between 316 and 380 in Altamira, Pacajá, Conceição do Araguaia, and Rio Maria (see the blue arrows in Figure 2b). A total of 5 of these 11 municipalities at the top of the ranking are in the southeast, 5 are in the center, and 1 is in the southwest of Pará. These regions can be considered the most critical in terms of the dengue transmission dynamics because the WHO considers an epidemic situation when the number of IDengue cases exceeds 300 cases per 100,000 inhabitants [20]. Comparatively lower incidence rates, between 50 and 100 and also 20 and 50, are found in the cities located further northeast in the state, including the coastline strip.

**Figure 3:**
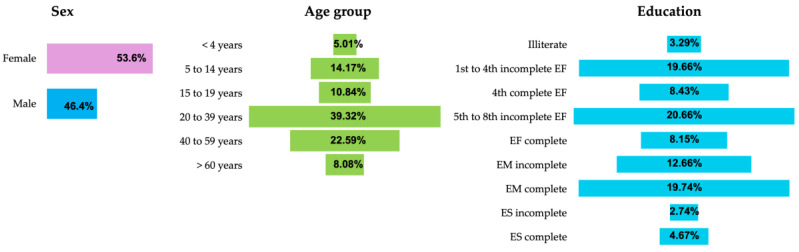
Epidemiological information for the eleven municipalities considered critical (>300 cases per 100,000 inhabitants) concerning sex, age group, and education (EF = elementary education; EM = high school; and ES = university education).

Another aspect that can be inferred from the updated dengue mapping is the social dimension in terms of its epidemiology. Comparing the data from the eleven municipalities considered to have the highest prevalence of dengue in the state, in relation to the total sample of 86, the contribution is around 19.6%. Therefore, it is relevant to report the information that characterizes these cities, which may be useful for priority public health initiatives in the region. Figure 3 presents the representative results of the eleven municipalities with critical dengue transmission. People with brown skin represent 74%, and the distribution by sex is slightly higher for women (54%). The age group most affected by the disease includes the adult population between 20 and 39 years old (39%) and also that between 40 and 59 years old (22%). In terms of education, dengue predominates in people from the two groups with an incomplete elementary education (1st to 4th grade and 5th to 8th grade), which total approximately 40%.

### 3.2. Relationships Between Climate Variables and Dengue Incidence

**Figure 4:**
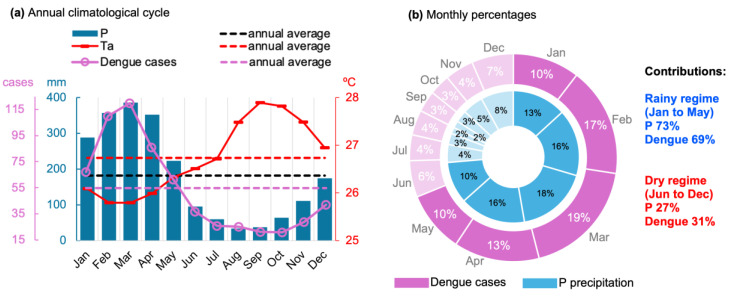
The climatological aspects representing the 86 municipalities: (**a**) the annual climatological cycle of P, Ta, and confirmed dengue cases; (**b**) the monthly percentages of P and dengue cases. The average P and Ta span from 1991 to 2020, while dengue spans from 2010 to 2024. Seasonal contributions are highlighted.

Determining the seasonal regimes in the study area was a prerequisite for evaluating the connection between climate factors and dengue in the state. Then, the monthly climatological evolution of P, Ta, and dengue cases representing the average of all 86 municipalities in Pará is analyzed in Figure 4a. A well-configured annual climatological cycle is observed in the region, with the annual average P (182 mm, the black dashed line) separating the rainiest period throughout the year, the months of January to May, with a peak of 386 mm reached in March. During this period, temperatures are milder, ranging from 25.8 to 26.3 °C. Dengue cases coincide with the rainfall pattern and present maximum values of 67, 110, 120, 86, and 62 cases in January, February, March, April, and May, respectively. On the other hand, in the remaining months of the year, the precipitation reaches its minimums between June and October, with rainfall reducing to 34 mm in August and 37 mm in September. During this period, air temperatures are significantly higher, peaking at almost 28 °C in September. These drier and warmer seasonal conditions are associated with fewer dengue cases, with only 21 cases in September and October. In the monthly percentages in Figure 4b, the five consecutive months with P above 10% are considered the RAINY regime: January to May. The percentages of dengue cases also show monthly values above 10% in the RAINY season. On the other hand, the months with P below 10% characterize the DRY season, from June to December. Analyzing the accumulated seasonal contribution, the five months of the RAINY regime explain 73% of the precipitation and 69% of the dengue cases in relation to the annual total. Therefore, this period is clearly the most relevant in terms of the incidence of arboviruses in the state of Pará. The seasonal patterns established by [26] using historical precipitation data gathered by a network of meteorological stations in eastern Amazonia are consistent with such regimes derived from the ERA5 data.

**Figure 5:**
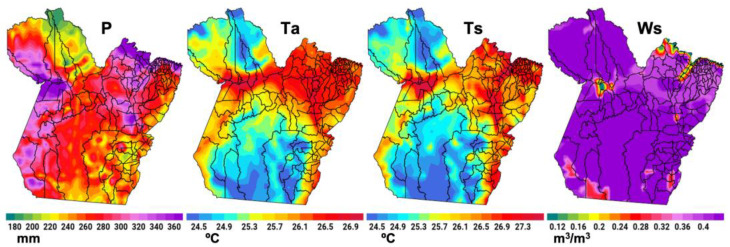
The 2010–2024 ERA5 average P, Ta, Ts, and Ws in the state of Pará in the RAINY regime.

The ERA5 seasonal mean maps are displayed in Figure 5 with the spatial configuration of the variables P, Ta, Ts, and Ws considering the RAINY regime in the period from 2010 to 2024, which encompasses the sample period for the IDengue data. The spatial configuration of P shows high spatial variability, with maximums above 300 mm in the western and northeastern portions. In the central–southern portion, rainfall varies between 240 and 280 mm, and the northern sector presents values below 220 mm. Ta and Ts show similar spatial patterns, with warmer regions along the western, central, and northeastern strip of the state, where values between 26.1 and 27.3 °C are observed. The soil’s moisture content is high, above 0.36 m^3^/m^3^ in most of the state of Pará.

**Figure 6:**
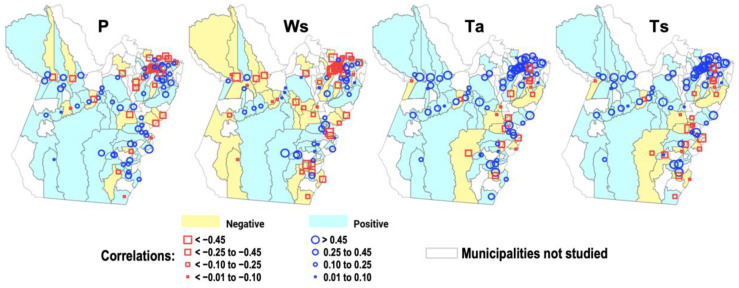
Maps of Pearson’s correlations between the RAINY regime climate variables (P, Ws, Ta, and Ts) and the IDengue annual series for the 86 municipalities of Pará. Municipalities in yellow indicate correlations < 0 and those in blue > 0.

Figure 6 and Table 1 show the results of the relationships between the seasonal series of climate variables and the annual series of IDengue for the 86 municipalities within the state of Pará. For the variable P, the group of municipalities containing negative correlations are mainly limited to the northeastern sector, with few individual points in the west and east portions, while the group with positive correlations is shown to extend across the state along the west, center, southeast, and southwest sectors. Comparing the P and WS maps, there is an increased number of municipalities in the negative correlation group throughout the state, mainly in the west, central, and southeast sectors, while the positive group is retained in the same regions but with smaller numbers of municipalities. Concerning the variables Ta and Ts, both maps present negative correlations in isolated locations in the southeast and east portions, while most of the state of Pará present a predominance of positive correlations.

In the results in Table 1, the number of municipalities and the corresponding percentages of the group with positive correlations for the P variable show comparatively higher values than those for the group with negative correlations, at 51 to 27 with proportions of 59% to 31%, respectively; these results show that the difference is statistically significant. For the variable Ws, the numbers between the positive and negative groups are close, and therefore there is no statistically significant difference. On the other hand, the results show notable differences between the groups for the climate variables Ta and Ts, connecting the soil’s thermal conditions and the atmosphere’s thermal conditions. The percentage in the positive group is 68% for Ta and 63% for Ts, with the chi-square test revealing statistically significant differences, according to a *p*-value < 0.05 being confirmed for these cases.

### 3.3. The Effects of the RAINY Regime on Dengue Incidence

**Figure 7:**
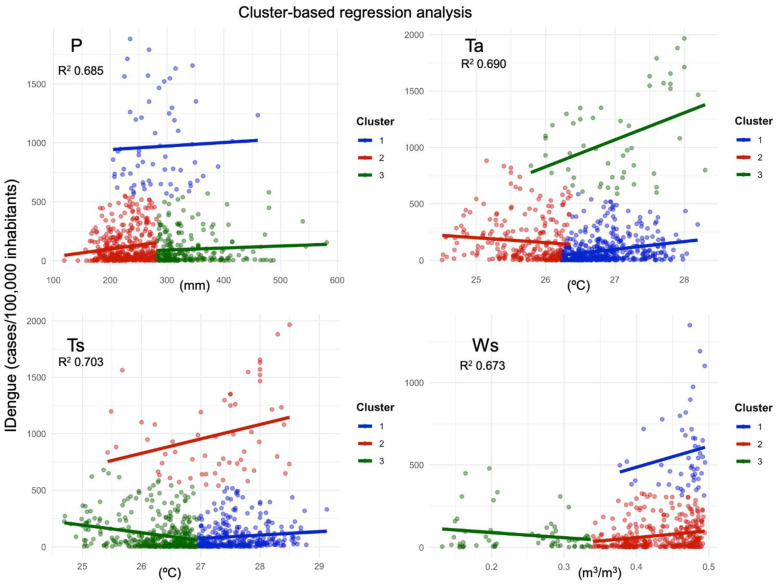
Scatter diagrams between the climate variables P, Ta, Ts, and Ws for the RAINY regime and IDengue obtained in the cluster-based regression analysis.

The results of the cluster-based regression analysis are shown in Table 2 and Figure 7, with the interpretation of the scatter diagrams for the climate variables and IDengue, the slope curves, and the distribution of points varying in their intensities on each axis. Table 2 provides the quantitative and statistical information obtained using the models for each climate variable.

The P diagram reveals positive slope curves for all clusters, i.e., precipitation has a direct and positive effect on contributing to an increase in the dengue incidence in the state of Pará, with the model explaining about 68.2% of the variability in the observations considering the differences between the clusters (different y levels, with different intercepts). Cluster 2 exhibits the greatest positive slope and the maximum intercept value, i.e., the effect of the climate is stronger in municipalities with precipitation patterns below 290 mm, associated with dengue rates below 500 cases/100,000 inhabitants. Lower influences are verified in cluster 1, characterized by municipalities with the highest dengue incidence rates (between 500 and 2000) and P ranging from 200 to 450 mm. Cluster 3, expressing locations with an incidence below 500 and P between 300 and 550 mm, shows the lowest slope and intercept. Concerning the variable Ws, with the model explaining 67.3% of the observed data, a stronger positive effect (a steep slope and the highest intercept value) is observed in the municipalities with the highest dengue rates (above 500), which are favored by the high availability of water in the soil Ws > 0.38 m^3^/m^3^ (cluster 1). For municipalities with IDengue below 500 cases/thousand inhabitants, an inflection point is observed at the threshold around 0.34 m^3^/m^3^. A soil water volume below this threshold has an inverse effect (a negative slope and intercept) in municipalities with IDengue < 500 (cluster 3), while soil moisture conditions above this threshold favor a systematic increase (a positive curve and intercept) in the disease in municipalities with dengue rates < 300 cases.

The R^2^ values obtained in the regression of the variables Ta and Ts reveal that the models explain approximately 69% and 70% of the observations, respectively. Analyzing the diagrams for Ta and Ts, a group with the most pronounced positive slope is evident, with particular reference to the municipalities characterized by the highest dengue rates in the state (IDengue between 500 and 2000 cases/100 thousand inhabitants). This group presents the highest intercept values (Table 2), so environmental conditions within the atmosphere of Ta > 25.8 °C (cluster 3) and within the soil of Ts > 25.5 °C (cluster 2) positively favor an increasing cycle of disease transmission. Furthermore, the regression results detected interesting, opposite effects, particularly in the groups of municipalities presenting low to medium IDengue (0 to 500 or up to 750 cases/100 thousand inhabitants). Under milder air and soil temperature conditions, of Ta < 26.2 °C (cluster 2) and Ts < 27.0 °C (cluster 3), negative slopes and intercepts are evidenced, thus indicating an unfavorable effect on the proliferation of dengue. Conversely, when the atmosphere and the soil are configured to have warmer conditions, of Ta > 26.2 °C (cluster 1) and Ts > 27 °C (cluster 1), there is a tendency towards a systematic increase in dengue transmission. So, these results show apparent inflection points in temperature of around 27 °C for the soil and 26.2 °C for the atmosphere for which variations above/below these thresholds determine positive/negative effects on dengue cases in the state.

**Figure A1:**
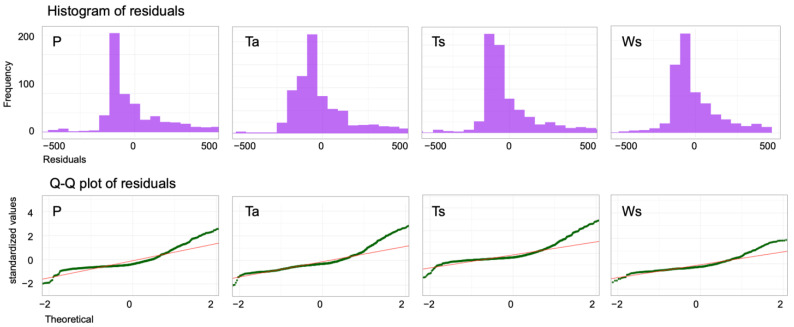
Histograms (top) and Q-Q plots (bottom) of residuals for each climate variable.

The analysis of the residuals in each model was based on the quantitative information in Table 2, alongside a careful visual inspection of the diagnostic plots (histograms and Q-Q plot of residuals) shown in Figure A1 (Appendix). In terms of the goodness-of-fit, high adjusted R^2^ values between 0.682 (for P) and 0.70 (for Ts) indicate that the models were able to capture a large portion of the variance in the dependent variable. The high F-statistic values between 279 (for Ws) and 363 (for Ts) with respective significant p-values demonstrate that the predictors x (climate variables) in the form of different clusters collectively have a significant relationship with the response variable y (dengue incidence). When comparing the residual plots and Q-Q plots for each variable (Figure A1, Appendix), we noticed that the models showed a few systematic patterns (i.e., the distribution of the residuals into an approximately normal curve shape and a Q-Q plot that closely followed a diagonal line), diagnostics of which revealed that the cluster-based regression analysis was successful and demonstrated a good statistical fit between the variables studied.

## 4. Discussion

In a retrospective study of the epidemiology of dengue in Brazil, Oneda [27] reported that in the northern region, 80% people with brown skin, 44% male and 56% female proportions, and a prevalence of 38% individuals aged between 20 and 39 years old acquire dengue. Analyzing the state of Amazonas, Paixão [28] found a greater incidence of dengue in adults aged between 20 and 29 years old and 30 and 39 years old, with equal proportions between males and females. The epidemiological results in the most critical areas of Pará (Figure 3) coincide with these previous studies. An interesting explanation for the higher prevalence in women is that in addition to them spending more time at home (prolonged exposure to mosquito habitats), they tend to seek health services more frequently and are therefore diagnosed more frequently [29].

Of relevance to the scope of this work are studies that have used historical data to address the association between the Amazonian climate (cities located in the states of Roraima, Acre, Rondônia, Amazonas, and Pará) and the rate of dengue incidence, whose findings using correlations [12,22,23,24] and other quantitative methods [11] are aligned with the results obtained in the present work. The statistical analyses corroborate this understanding of the complex dynamic cycle of dengue transmission. The direct relationship (positive correlation patterns) between rainfall and dengue can be interpreted as the contribution of periods of heavy rainfall to increasing the number of breeding sites (soil surfaces and containers that accumulate stagnant water) for mosquitoes and thus favoring the development of these vectors in the aquatic phase [7,8,30]. Here, we demonstrate for the eastern Amazon that this association is maximized seasonally in the rainy season, with the incidence of dengue corresponding to 69% (relative to the annual total) in just five months (January to May, Figure 4) when the regional climate conditions favor the proliferation of the arbovirus. The cluster-based regression analysis approach detected some particularities in the effects of climate on dengue fever throughout the state of Pará (Figure 7). In general, the P variable contributes directly and positively to the disease, i.e., increased rainfall means an enhanced incidence of dengue, but this effect is stronger in municipalities with rainfall patterns below 290 mm, associated with incidence rates below 500 cases/100,000 inhabitants. For the variable Ws, related to the availability of water in the soil for breeding sites of the vector mosquito, the regression analysis demonstrated an important result for municipalities with a dengue incidence below 500 cases/100,000 inhabitants, indicating that there is an inflection point at around 0.34 m^3^/m^3^. Under soil moisture conditions below/above this threshold, there is a negative/positive effect on disfavoring/favoring the intensification of dengue cases in the region. This is a noteworthy result that has not yet been explored in previous studies, and here, it was possible to address using the ERA5 reanalysis.

On the other hand, the effect of temperature on the environment in which the dengue transmission cycle occurs is also relevant. The linear relationship of the positive correlation patterns between thermal conditions and dengue cases was expected, with the explanation that higher temperatures contribute to the different stages of the mosquito’s development cycle, including maturation, survival, and population growth, as well as favoring hematophagic activity and viral replication [31,32]. In a continental-scale modeling study, Mordecai [10] demonstrated that the magnitude of dengue’s transmission (and also Zika and chikungunya transmission) by the two most common types of vector mosquito (*Aedes aegypti* and *Ae. Albopictus*) changes consistently with the average surface temperature. The tropics and subtropics have temperatures suitable for vector development throughout the year, but the maximum transmission occurs in months with a thermal range between 26 and 29 °C. Under these conditions, temperature accelerates larval development, making the mosquito life cycle more active (reducing the extrinsic incubation time of the virus) and leading to more bites on exposed humans, thus contributing to a higher efficiency in the dengue transmission rate [33,34]. The correlation maps for the Ta and Ts variables presented in this study showed that the positive group is proportionally (statistically significantly) higher than the negative one (Figure 6, Table 1), evidencing the direct relationship between air and soil temperature and dengue cases in most municipalities in Pará (eastern Amazon). The regression analyses were able to explain the differentiated effects of temperature on the different clusters of dengue incidence across the state (Figure 7). It was clear that there is a stronger modulation of soil and air temperature in municipalities where dengue transmission is more critical (with rates between 500 and 2000 cases/100 thousand inhabitants). A relevant result was evidenced in the municipalities presenting low- to moderate-intensity dengue rates (between 0 and 500), where the thermal conditions of the soil, with a threshold of around 27 °C, and of the atmosphere, with a threshold of around 26.2 °C, can determine the positive or negative effect on the proliferation of the disease throughout the state. These values are consistent with previous studies that have analyzed air temperature [33,34], but here, it was possible to indicate the effect of the soil based on the Ts data from ERA5. With this, our scientific knowledge about the effect of temperature on the mosquito’s development cycle became more complete, i.e., under thermal conditions in the soil above 27 °C and in the air above 26.2 °C, there are amplification effects on the dynamics of dengue transmission in the region.

## 5. Conclusions

The state-wide transmission of arboviruses was shown through updated mapping of positive dengue cases that have officially been registered across Pará (eastern Brazilian Amazon) during the last 15 years (2010 to 2024). However, there are areas where the average dengue incidence is critical, primarily in the central, southeast, and southwest regions. Eleven municipalities have extreme dengue counts exceeding 300 cases per 100,000 inhabitants, which the World Health Organization considers to be epidemic conditions. In terms of decision-making and the development of public health policies for the Amazonian population, the mapping’s findings and the epidemiological data produced by this study are pertinent.

This study provides evidence that regional climate variables play a critical and spatially differentiated role in modulating dengue transmission across the eastern Brazilian Amazon, particularly in the state of Pará. The findings reveal that dengue cases predominantly occur during the rainy season (January to May), accounting for 69% of annual cases, when climatic conditions maximize vector proliferation. The cluster-based regression analysis showed that precipitation has a stronger effect in municipalities with rainfall below 290 mm, while soil moisture exhibited a critical threshold at 0.34 m³/m³, determining either a suppressing or amplifying effect on dengue incidence. Furthermore, air temperature (above 26.2 °C) and soil temperature (above 27 °C) were found to be pivotal in enhancing transmission, particularly in regions with moderate to high incidence rates (500–2000 cases per 100,000 inhabitants). These results not only align with previous studies on climate–dengue relationships but also provide novel insights, such as the influence of soil temperature, a previously underexplored factor. Thus, this research advances the understanding of climatic drivers of dengue in the eastern Amazon, which combined with other socio-environmental factors in the exposed population, offering valuable evidence for region-specific surveillance and epidemiological control strategies.

## Figures and Tables

**Table 1:** The number (count) of municipalities in each group of positive (≥0.10) and negative (≤−0.10) correlations for each variable and the respective proportion (in relation to the entire sample = 86) and the results of the chi-square test.

Groups	Results	P	Ws	Ta	Ts
Group of positive correlations	count	51 *	30	59 *	57 *
Group of negative correlations		27	37	12	16
Group of positive correlations	proportion	59.3%	34.9%	68.6% *	63.3% *
Group of negative correlations		31.4%	43.0%	14.0%	18.6%
Group of positive correlations	chi-square	7.38 *	0.73	31.11 *	23.03 *
Group of negative correlations	*p*-value	0.007	0.392	0.000	0.001

* Statistically significant values (a *p*-value < 0.05).

**Table 2:** Quantitative and statistical information obtained in the cluster-based regression models for each climate variable.

Variables	R^2^	Adjusted R^2^	Residual Std Error	F-Statistic	Intercept		
					Cluster 1	Cluster 2	Cluster 3
P	0.685	0.682	167.1	304.7 *	1.30	2.67	0.97
Ws	0.673	0.669	130.4	279.9 *	979.5	406.1	−306.1
Ts	0.703	0.701	154.9	363.6 **	29.7	127.5	−66.6
Ta	0.690	0.688	163.7	351.4 **	69.1	−43.2	239.5

Statistically significant values. ** *p*-value < 0.05 and * *p*-value < 0.10.

## Data Availability

The databases and their respective sources and references were described in the Materials and Methods section.
